# Association Between Proteomic Blood Biomarkers and DTI/NODDI Metrics in Adolescent Football Players: A Pilot Study

**DOI:** 10.3389/fneur.2020.581781

**Published:** 2020-11-16

**Authors:** Keisuke Kawata, Jesse A. Steinfeldt, Megan E. Huibregtse, Madeleine K. Nowak, Jonathan T. Macy, Kyle Kercher, Devin J. Rettke, Andrea Shin, Zhongxue Chen, Keisuke Ejima, Sharlene D. Newman, Hu Cheng

**Affiliations:** ^1^Department of Kinesiology, School of Public Health-Bloomington, Indiana University, Bloomington, IN, United States; ^2^Program in Neuroscience, College of Arts and Sciences, Indiana University, Bloomington, IN, United States; ^3^Department of Counseling and Educational Psychology, School of Education, Indiana University, Bloomington, IN, United States; ^4^Department of Applied Health Science, School of Public Health-Bloomington, Indiana University, Bloomington, IN, United States; ^5^Division of Gastroenterology and Hepatology, Department of Medicine, Indiana University School of Medicine, Indianapolis, IN, United States; ^6^Department of Epidemiology and Biostatistics, School of Public Health-Bloomington, Indiana University, Bloomington, IN, United States; ^7^Department of Psychological and Brain Sciences, College of Arts and Sciences, Indiana University, Bloomington, IN, United States; ^8^Alabama Life Research Institute, University of Alabama, Tuscaloosa, AL, United States

**Keywords:** brain injury, diffusion tensor imaging, neurite orientation dispersion and density imaging, blood biomarker, football, youth, concussion, subconcussion

## Abstract

While neuroimaging and blood biomarker have been two of the most active areas of research in the neurotrauma community, these fields rarely intersect to delineate subconcussive brain injury. The aim of the study was to examine the association between diffusion MRI techniques [diffusion tensor imaging (DTI) and neurite orientation/dispersion density imaging (NODDI)] and brain-injury blood biomarker levels [tau, neurofilament-light (NfL), glial-fibrillary-acidic-protein (GFAP)] in high-school football players at their baseline, aiming to detect cumulative neuronal damage from prior seasons. Twenty-five football players were enrolled in the study. MRI measures and blood samples were obtained during preseason data collection. The whole-brain, tract-based spatial statistics was conducted for six diffusion metrics: fractional anisotropy (FA), mean diffusivity (MD), axial/radial diffusivity (AD, RD), neurite density index (NDI), and orientation dispersion index (ODI). Five players were ineligible for MRIs, and three serum samples were excluded due to hemolysis, resulting in 17 completed set of diffusion metrics and blood biomarker levels for association analysis. Our permutation-based regression model revealed that serum tau levels were significantly associated with MD and NDI in various axonal tracts; specifically, elevated serum tau levels correlated to elevated MD (*p* = 0.0044) and reduced NDI (*p* = 0.016) in the corpus callosum and surrounding white matter tracts (e.g., longitudinal fasciculus). Additionally, there was a negative association between NfL and ODI in the focal area of the longitudinal fasciculus. Our data suggest that high school football players may develop axonal microstructural abnormality in the corpus callosum and surrounding white matter tracts, such as longitudinal fasciculus. A future study is warranted to determine the longitudinal multimodal relationship in response to repetitive exposure to sports-related head impacts.

## Introduction

Concussive and subconcussive brain injury in sports have emerged as a complex public health issue. Policy and rule changes, as well as societal awareness, have played a catalytic role in decreasing concussion incidence in sports (1). However, despite decades of investigation, there is no concrete evidence on gold-standard diagnostic biomarkers for concussion, preventive tools that can increase neural resiliency to trauma, or factors contributing to the potential long-term consequence of subconcussive head-impact exposure. This knowledge gap is partly due to the unimodal approach, in which many papers report data derived from a single modality (e.g., neuroimaging, blood biomarker, behavioral measures). This precludes validation of study findings. For example, elevated tau protein in blood theoretically indicates axonal damage or degeneration, but without cross-referencing against neuroimaging data, the usefulness of the tau protein as a surrogate for brain damage remains speculative at best.

Several interdisciplinary groups have begun testing two- and three-way multimodal relationships that reflect subconcussive neuronal stress. In 2014, initial studies by Talavage et al. (2) and Bazarian et al. (3) revealed head impact-dependent declines in neural activation patterns and axonal microstructural integrity after a single high school and college football season, respectively. These neuroimaging findings were correlated with declined cognitive function (2, 4), and the development of autoimmune response to brain-derived blood biomarkers (e.g., ApoA1, S100B) (3, 5). Despite the unequivocal importance of the multimodal approach, association studies in subconcussion research are limited (6, 7).

Neuroimaging techniques, especially diffusion MRI, and brain-derived blood biomarkers are the fastest growing areas of neurotrauma research. Diffusion tensor imaging (DTI) is the most extensively used technique worldwide to examine the white matter microstructural properties in humans. However, DTI metrics such as mean diffusivity (MD) and fractional anisotropy (FA) represent basic statistical descriptions of diffusion that do not directly correspond to biophysical properties of neuronal axons (8). In 2012, Zhang et al. (9) introduced the neurite orientation and dispersion density imaging (NODDI) technique that can measure axonal density within white matter, dispersion of axonal orientation, and free water diffusion (see Table 1 for descriptions of these metrics). The combined use of DTI and NODDI has been shown to detect progressive axonal degeneration even 6 months after a concussion (13). Similar to neuroimaging techniques, blood biomarker technology has evolved to be able to detect neural factors at a femtomolar concentration. Among the many potential biomarkers for brain injury, tau, neurofilament-light (NfL), and glial fibrillary acidic protein (GFAP) have shown their superior ability to predict concussion recovery time (30, 31), cumulative subconcussive axonal damage (26, 32, 33), and absence of intracranial bleeding (34–36). However, the relationships between DTI/NODDI metrics and blood biomarkers in reflecting cumulative neural stress from football head impacts have never been reported in the literature.

**Table 1:** Summary of DTI/NODDI and blood biomarker characteristics.

**Outcome metrics**	**Definition**	**Cause of increase**	**Cause of decrease**
Fractional anisotropy (FA)	A precise assessment of white matter microstructure properties like myelination, packing density, axonal coherence, axonal size, and microstructural connectivity, which is characterized by the directionality of constrained water diffusion in the brain tissue (10–12).	- Increase found following repetitive subconcussive hits over long periods of time, conflating injury, and recovery effects (13). - Athletes found to have an elevation with a history of concussions, noting hindered water diffusion within white matter tracts (14).	- A lower level of FA observed 2 weeks post-injury may be due to an influx of water content as a response to neuroinflammation (13). - In cases of more severe TBI, a decrease of FA is generally noted as individuals display more clinical symptoms. The persistent microstructural changes noted with concussions may be more distinguishable from severe TBI (14).
Mean diffusivity (MD)	The average rate of molecular diffusion measured from all directions with the assumption cellular size and integrity play a role (15, 16).	- An increase following 2 weeks post-injury may be due to an influx of water content as a response to neuroinflammation (13). - Increase is observed in individuals who experience a more severe form of TBI (14).	- A decrease was noted in athletes with a history of concussion, due to a hampering of water diffusion within white matter tracts (14). - Following a concussion, during the post-injury phase, MD was noted to be decreased (17).
Axial diffusivity (AD)	The rate at which water molecules diffuse parallel to the tract within the voxel of interest (18).	- Following repetitive hit hockey season an increase in AD was noted (19).	- Decrease noted 24 h post-concussion (17).
Radial diffusivity (RD)	The immensity of water molecule diffusion occurring perpendicular to the tract within the voxel of interest (18).	- Following repetitive hit hockey season an increase in RD was noted (19).	- Decrease noted 24 h post-concussion (17).
Neurite density index (NDI)	The volume and density of neurites within intra-neurite space (20, 21).	- An increase is found after repetitive subconcussive hits over an extended period of time, prolonging injury and recovery effects (13).	- Decrease is observed over time in both initial and replication of mTBI suggesting progressive axonal degeneration (13). - A decrease is noted in NDI following a mTBI compared to orthopedic trauma controls and friend controls (13).
Orientation dispersion index (ODI)	Assess the characteristics of neurite angular variation of extra-neurite space as well as cell membranes, somas, glial cells, and fiber orientations in white matter (20, 21).	- Following mTBI, ODI was noted to be higher when observed in lower functioning individuals (13).	- ODI was noted to be lower in patients who did not show symptomatic or cognitive improvement following mTBI (13). - Concussed athletes displayed a decrease of ODI (14).
Tau	A microtubule binding protein in which promotes polymerization and plays a role in maintaining azonal transport and neuronal integrity (22).	- Noted to be elevated following high head impact pregame vs. post-game and preseason vs. post-season (23).	N/A
Neurofilament-light (NfL)	Protein associated with neuronal cytoskeletal element, which is involved in normal axonal and dendritic structure, growth, and function (24).	- Exposure to repetitive subconcussive head trauma results in an increase of NfL and remains elevated throughout the season (25). - Higher frequency and magnitude of head impacts results in an increase of NfL (24). - Following a bout of 10 subconcussive soccer heading impacts, NfL gradual increased, which was detected 2 h post-impact (26).	N/A
Glial fibrillary acidic protein (GFAP)	The most abundant cell type in the brain responsible for supporting structural integrity of the astrocytic cytoskeleton following disruptive forces (27).	- The severity of the TBI is dependent upon the rise in GFAP (28). - Within 1 h of a concussion, GFAP levels were noted to be increased, and reaching a peak level at 20 h post-injury (29).	N/A

Therefore, we conducted a pilot, cross-sectional study in high school football players to examine the relationship between diffusion neuroimaging metrics and blood biomarkers at their preseason baseline, aiming to detect potential residual neuronal damage from prior football seasons. We hypothesized that there would be significant associations between neuroimaging and blood biomarkers to reflect axonal microstructural damage in some areas of the brains of football players. Specifically, tau and NfL levels will correlate with higher (worse) levels in DTI and NODDI metrics (e.g., FA, MD, NDI), whereas GFAP will not show a notable correlation with DTI and NODDI metrics.

## Materials and Methods

### Participants

This single-site, cross-sectional study enrolled 25 male high school football athletes. None of the 25 participants was diagnosed with a concussion or traumatic brain injury in the 12 months prior to the enrollment. Inclusion criterion was being an active high school football team member. Exclusion criteria included a history of head and neck injury, including concussion within 12 months prior to the study or history of neurological disorders. However, participants were allowed to have a history of concussion if it was beyond 12 months prior to the study. Conditional exclusion criteria for the neuroimaging data collection were metal implants in the body or implanted electro/magnetic devices (e.g., orthodontic braces, pacemakers, aneurysm clips). The Indiana University Institutional Review Board approved the study, and all participants and their legal guardians gave written informed consent. The data were collected during the preseason baseline assessment in July 2019 and included self-reported demographic information (age, race/ethnicity, height, weight, number of previously diagnosed concussions, and years of tackle in American football experience), 7 mL of blood samples, and MRI scans.

### Blood Biomarker Assessments

Seven-milliliter samples of venous blood were collected into red-cap serum vacutainer sterile tubes (BD Bioscience). Blood samples were allowed to clot at room temperature for a minimum of 30 min. Serum was separated by centrifugation (1,500 × g, 15 min) and stored at −80°C until analysis. Serum levels of tau, NfL, and GFAP were measured using the Simoa^TM^ Platform (Quanterix), a magnetic bead-based, digital enzyme-linked immunosorbent assay (ELISA) that allows detection of proteins at femtomolar concentrations (37). An analytical protocol was previously described in detail (38). The analyses were performed by a board-certified laboratory technician blinded to the study design and subject characteristics. Limit of detection was 0.024 pg/mL for tau, 0.104 pg/mL for NfL, and 0.221 pg/mL for GFAP. The average intra-assay coefficients of variation for the samples were 6.7 ± 5.2% for tau, 8.3 ± 6.0% for NfL, and 3.7 ± 2.7% for GFAP.

### MRI Acquisition

The MRI data were acquired on a 3T Siemens Prisma MRI scanner (Siemens, Erlangen, Germany) equipped with a 64-channel head/neck coil. High-resolution anatomical images (T1 weighted) were acquired using 3D MPRAGE pulse sequence with the following parameters: repetition time/echo time (TR/TE) = 2,400/2.3 ms, inversion time (TI) = 1,060 ms, flip angle = 8, matrix = 320 × 320, bandwidth = 210 Hz/pixel, iPAT = 2, resulting in 0.8 mm isotropic resolution. For diffusion analysis, two consecutive diffusion weighted imaging (DWI) sessions with opposite phase encoding directions were performed with a simultaneous multi-slice single-shot spin-echo echo-planar pulse sequence with the following parameters: TE = 89.4 ms; TR = 3,590 s, flip angle = 90, 1.5 mm isotropic resolution. Each session had 103 images with different diffusion weightings and gradient directions summarized as following: 7 b = 0 s/mm^2^, 6 directions with b = 500 s/mm^2^, 15 directions with b = 1,000 s/mm^2^, 15 directions with b = 2,000 s/mm^2^, and 60 directions b = 3,000 s/mm^2^.

### Imaging Processing

First, the DWI images were denoised using the principal component analysis (PCA)-based denoising tool in Mrtrix (https://www.mrtrix.org/) (39), and then magnetic field map information for susceptibility artifacts correction was derived from the b0 (b = 0 s/mm^2^) images with opposite phase encoding directions using “topup” tool in FSL (https://fsl.fmrib.ox.ac.uk/fsl/fslwiki) (40). The images were then corrected for susceptibility artifact, eddy current distortions, and motion artifacts simultaneously using the “eddy” command of FSL and the average of the b0 volumes as a reference. DTI analysis was performed using the FSL Diffusion Toolbox. The diffusion metrics of FA, MD, axial diffusivity (AD), and radial diffusivity (RD) maps were calculated.

Meanwhile, the NODDI metrics including neurite density index (NDI) and orientation dispersion index (ODI) were derived using the NODDI Matlab toolbox v1.01 (http://mig.cs.ucl.ac.uk/index.php?n=Tutorial.NODDImatlab) using the default settings. NDI primarily represents axonal density within white matter, and ODI represents organization of white matter tracts (13).

### Statistical Analysis

The whole-brain, tract-based spatial statistics was conducted for six diffusion metrics: FA, MD, AD, RD, NDI, and ODI in FSL (41). The FA maps were co-registered to a template *via* non-linear transformation. A skeleton of mean white matter tracts was obtained, and FA values of nearby voxels were projected to the template to obtain skeletonized FA maps. The non-linear warps and skeleton projection derived from FA maps were applied to all other diffusion metrics to obtain skeletonized maps of MD, AD, RD, NDI, and ODI as well.

The analysis used complete samples to test the relationship between blood biomarker and MRI data. Univariate regression analyses were conducted for each diffusion metric against blood biomarker levels *via* randomized permutation. The model included years of tackle football experience and number of concussion occurrences as covariates. The Threshold-Free Cluster Enhancement (TFCE) option was used in the permutation test, which gives cluster-based thresholding for familywise error correction (42). As a result, the TFCE *p*-value images obtained were fully corrected for multiple comparisons across space. When there was a significant association, *post-hoc* analysis using a Pearson correlation coefficient was computed between the blood biomarker level and an average value of the imaging voxels that showed a significant effect in the regression analysis.

## Results

### Demographics

Five of 25 participants in the football group were excluded from MRI due to a metal implant in the body (*n* = 4) and orthodontic braces (*n* = 1). Three serum samples in the football group were not assessed for biomarkers due to severe hemolysis, which has shown to influence the detection of biomarkers, especially NfL (43). As a result, 17 completed sets of the neuroimaging-blood biomarker data for the analysis. Demographic information is detailed in Table 2.

**Table 2:** Demographics and biomarker levels.

**Variables**	**Football**
*n*	17
Sex (%)	17M (100)
Age, y	16 (16–17)
BMI, kg/m^2^	26.5 (23.9–28.7)
No. of previous concussion
0, *n* (%)	11 (65.0)
1, *n* (%)	5 (29.0)
2, *n* (%)	1 (6.0)
Tackle football experience, y	7 (3–8)
Race, *n* (%)
White	14 (82)
Black/African American	0 (0)
Asian	0 (0)
African Indiana/Alaska	0 (0)
Multiracial	3 (18)
Ethnicity, *n* (%)
Not Latino/Hispanic	14 (82)
Latino/Hispanic	3 (18)
Psychiatric condition
ADHD	0 (0)
Learning disability	0 (0)
Major depressive disorder	0 (0)
Blood biomarker levels, mean±SD, pg/mL
Tau	2.26 ± 1.10
Neurofilament light	4.27 ± 1.89
Glial fibrillary acidic protein	60.00 ± 22.04

*Data for age, BMI, and football experience are expressed as median (interquartile range), as these data were not normally distributed. Blood biomarker data were normally distributed; hence, it is presented as mean ± standard deviation. BMI, body mass index. ADHD, attention-deficit/hyperactivity disorder*.

### Associations Between Diffusion Metrics and Blood Biomarker Levels

**Figure 1:**
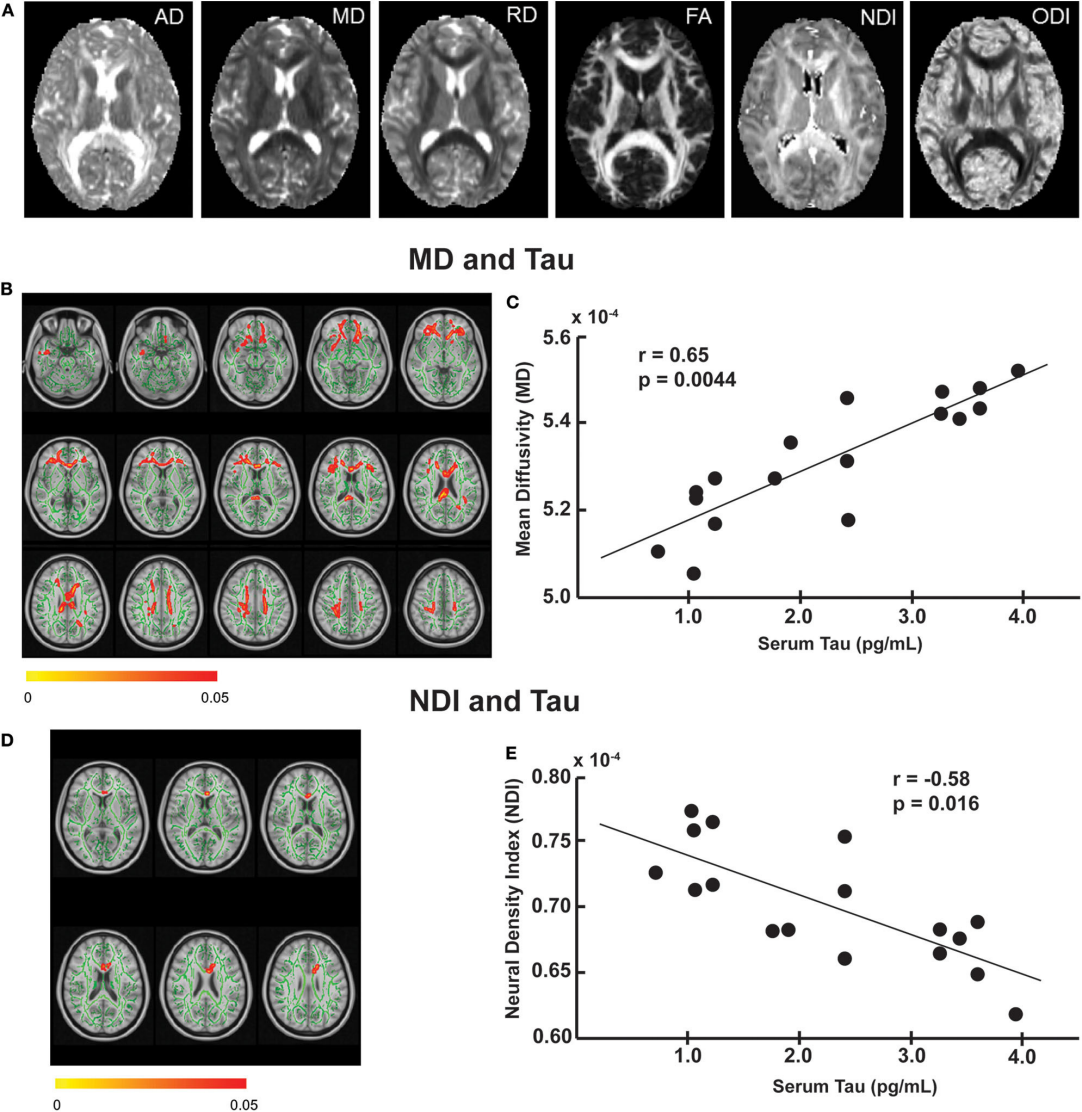
The relationship between imaging and serum tau levels. **(A)** Example maps of DTI [axial diffusivity (AD), mean diffusivity (MD), radial diffusivity (RD), fractional anisotropy (FA)] and NODDI [neurite density index (NDI), and neurite orientation dispersion index (ODI)] from a single subject in FMRIB58_FA template. Tract-based regression analysis showed that tau was positively associated with mean diffusivity **(B)** and negatively associated with neurite density index **(D)** in several white matter tracts (red-yellow, corresponding to *p*-value 0.05 to 0). The regression analyses were performed on the skeletonized white matter tracts (green) derived from the mean FA maps of the study subjects, to which diffusion metrics of nearby voxels were projected. All results were corrected for multiple comparisons using threshold-free cluster enhancement (TFCE) at *p* ≤ 0.05. The results are overlaid on the 1-mm resolution MNI (Montreal Neurological Institute) template. *Post hoc* correlation analysis revealed a positive correlation between tau and mean MD of the voxels that showed a significant effect in the regression analysis **(C)** and a negative correlation between tau and mean NDI of the voxels that showed a significant effect in the regression analysis **(E)**.

Examples of the six different diffusion metrics are shown in Figure 1A on one slice of a representative subject that is mapped on FMRIB58_FA standard space. These parameter maps are distinct from one another, with each metric (FA, MD, AD, RD, NDI, and ODI) characterizing different diffusion features arising from underlying tissue microstructure.

Tract-based regression analyses revealed significant associations between several diffusion metrics and blood biomarker levels in football players. Specifically, serum tau level was positively associated with MD (Figure 1B) and negatively associated with NDI (Figure 1D) mainly in the corpus callosum. The tau-MD association was widespread over the corpus callosum (*p* = 0.027) and longitudinal fasciculus, whereas the tau-NDI association was focal on the anterior body of the corpus callosum (*p* = 0.048). In our *post-hoc* analysis using a Pearson correlation coefficient, we found a significant positive correlation between an average value of MD voxels that showed significant associations in Figure 1B and serum tau levels (*r* = 0.65, *p* = 0.0044: Figure 1C). Similarly, there was a significant negative correlation between an average value of NDI voxels that showed significant associations in Figure 1D and serum tau levels (*r* = −0.58, *p* = 0.016: Figure 1E). All *post-hoc* comparisons are shown in Supplementary Table 1. It is worth noting that both covariates, years of tackle football experience, and number of previous concussions had a non-significant influence on the imaging-blood biomarker association.

**Figure 2:**
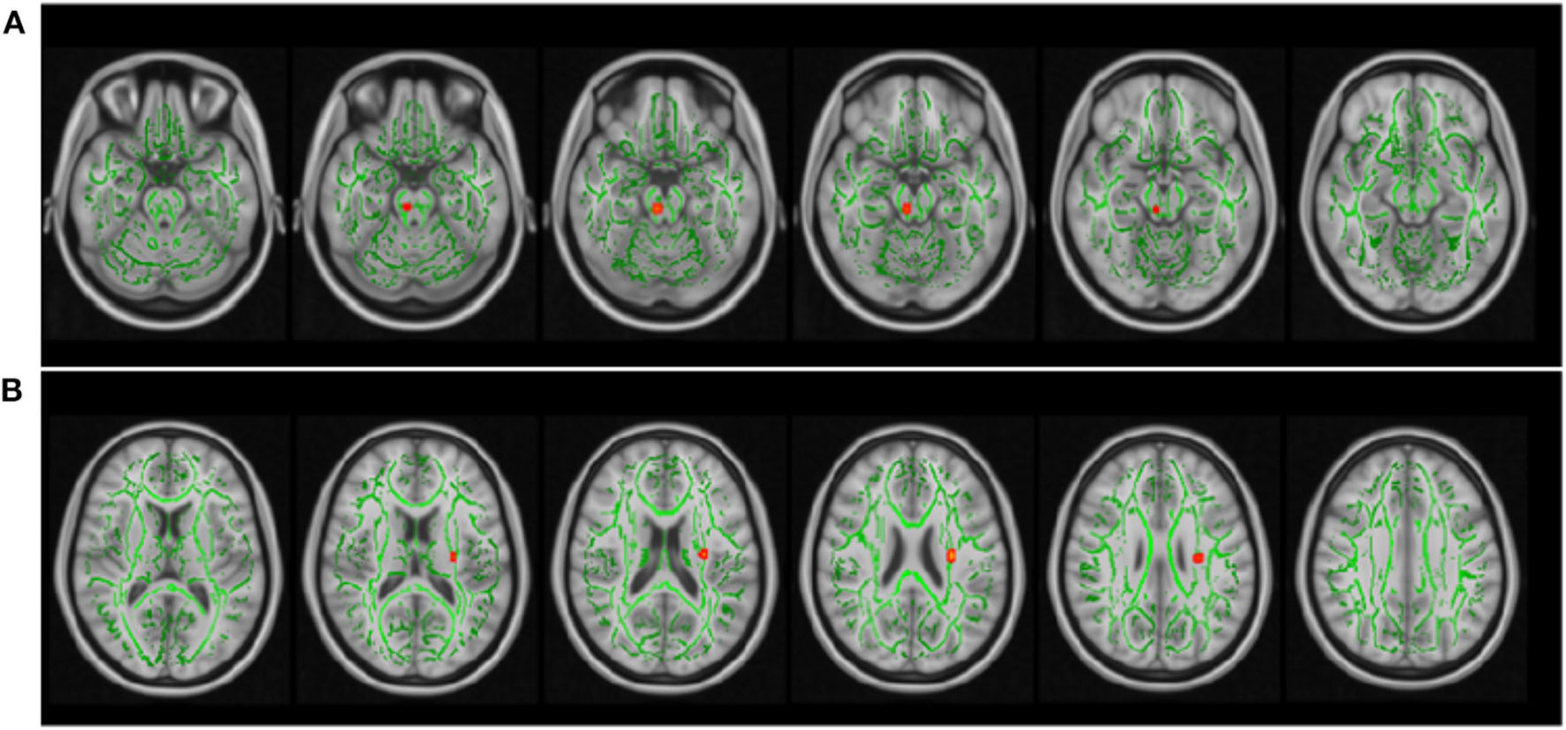
The relationship between imaging and serum GFAP and NfL levels. The same tract-based regression analysis as used in Figure 1 showed that GFAP was positively associated with axial diffusivity in the brain stem **(A)**, and NfL was negatively associated with neurite orientation dispersion index **(B)** in a small portion of the longitudinal fasciculus. The skeletonized white matter tracts (green) are the same as that in Figure 1. The TFCE *p*-value was set to *p* ≤ 0.05. The results are overlaid on the 1-mm resolution MNI template.

Additionally, a small number of voxels showed positive association between GFAP and AD in the brain stem (*p* = 0.052: Figure 2A) and negative association between NfL and ODI in the longitudinal fasciculus (*p* = 0.046: Figure 2B).

## Discussion

The novelty of the current study was the multimodal association of the neural injury blood biomarkers (tau, NfL, and GFAP) and diffusion imaging metrics derived from DTI and NODDI to gauge the potential cumulative stress in the brains of high school football players. Primary findings from this pilot study are that elevated serum tau levels at preseason baseline were reflective of increased MD in various white matter tracts and decreased neurite density in the corpus callosum. However, these associations were independent from a self-reported number of previous concussions and years of tackle football experiences.

The corpus callosum is comprised of nearly 200 million myelinated axonal tracts that enable interhemispheric neuronal communications (44). The corpus callosum has been shown to be one of the most vulnerable areas of the brain to concussive and subconcussive mechanical forces (e.g., shear, stretch, shortening) (45–47), and significant atrophy has been found in brains with chronic traumatic encephalopathy (48, 49). Furthermore, the immature (teenage) brain has shown to exhibit more pronounced axonal diffusion in the corpus callosum and longitudinal fasciculus (50, 51) following repetitive subconcussive head impacts compared to the mature brain (52), given that neural networks in the frontal cortex proliferate throughout adolescence and reinforce executive functions (53).

Our data on the imaging-blood biomarker associations in the corpus callosum and longitudinal fasciculus are intriguing, in that even at preseason baseline, increased MD and reduced NDI in these tracts were highly correlated to serum levels of tau in high school football players. The interpretation of DTI imaging data on subconcussive neurotrauma is challenging, as a recent systematic review (54) concluded that there are divergent findings in DTI measures. As for MD, it is thought that increased MD often attributes to more severe form of traumatic brain injury (TBI), whereas decreased MD is frequently reported due to repetitive subconcussive head impacts, as revealed in a recent systematic review (54). Given that our data is cross-sectional and we do not possess head impact kinematic data from previous seasons, it remains unclear of the cumulative head impact effects on the MD values. However, we identified that elevated MD in various axonal tracts was correlated strongly to elevated serum tau, which has shown to gauge the severity of axonal damage and neurodegenerative progression, including diagnosis of Alzheimer's disease (55), prediction of concussion recovery duration (30, 31), and association with short- (23) and long-term subconcussive neural stress (56).

This MD-tau finding is further substantiated by NDI data derived from NODDI analysis. NDI is a measurement of the intracellular volume fraction; in other words, NDI primarily represents axonal density within white matter. Palacios et al. (13) previously reported that concussion can significantly and acutely reduce NDI, and NDI values continues to decrease over time in these concussion patients, suggesting progressive axonal degeneration. We found a significant negative correlation between serum tau and NDI particularly in the corpus callosum. It is possible that football-related head impacts can trigger microstructural disruption and progressive degeneration in axons, as represented in elevated MD and reduced NDI, and concurrently induce tau dissociation from microtubules. Dissociated tau can reach peripheral circuitry through either blood–brain barrier leakage or glymphatic pathway (57). These associations are physiologically reasonable and indicate that playing American football may relate to chronic microstructural abnormality in axonal tracts. Previous studies support this hypothesis in such a way that astrocyte-enriched protein, S100B, is released into the peripheral circuitry in a subconcussive impact-dependent manner in college (58) and high school football players (5, 59). Recurring spikes of plasma S100B levels due to head impacts from practices and games can develop autoimmune reaction to S100B within neurons and astrocytes (5). Furthermore, abundant S100B in the brain parenchymal space can act as a ligand for the advanced glycation end products receptors in the neuronal plasma membrane (60), which then trigger a cascade of events including c-Jun N-terminal kinase, Dickkopf-1, and glycogen synthase kinase 3β, which collectively induce hyperphosphorylation of the tau protein and contribute to tau tangle formation (57). While it is unlikely to observe distinct neurodegenerative features in adolescent football players, a recent study suggests that subconcussive head impact exposure may blunt positive neurologic effects (i.e., increased axonal integrity, better cognitive performance) from participating in sports. Strauss et al. (61) demonstrated that these beneficial effects were absent in soccer players who experienced high exposure to soccer headings, pointing to the possibility that axonal microstructural damage from head impacts may attenuate neurologic well-being as part of healthy development in adolescents.

Similar to tau protein, NfL functions as a scaffolding structural protein in axonal and dendritic branching and growth, and NfL undergoes post-translational modification by a series of phosphorylation events, which can make it vulnerable to mechanical stretch and shear stress (62). Growing evidence supports that NfL levels in blood have shown to reflect the progression of neurodegenerative condition (e.g., Alzheimer's disease, multiple sclerosis) (63–65), differentiate severities of TBI (66), predict clinical outcome (e.g., functional recovery, return-to-play) after severe TBI and concussion (67, 68), and correlate with subconcussive head impact exposure (24, 26, 32, 33). Unlike tau, however, we failed to observe significant correlations between NfL and diffusion metrics, which opposes the data by Ljungqvist et al. (69) that showed an association between serum NfL and FA (*R*^2^ = 0.83) 12 months after severe TBI. This discrepancy may attribute to the severity of injury, whereby despite repeated exposure to subconcussive head impacts in previous seasons, these stimuli may not be sufficient to chronically elevate NfL levels. In fact, Joseph et al. (23) reported that serum NfL was unchanged after a high school football season, while tau protein elevated up to five-fold post-season, especially in players with frequent head impacts. A multimodal longitudinal study is needed to address whether serum levels of tau and NfL elevate due to subconcussive head impacts over time and associate with progressive axonal microstructural damage, as assessed *via* DTI/NODDI.

### Limitations

While the current study used state-of-the-art technologies to examine the brain microstructural integrity of adolescent athletes, there were limitations to be noted. A relatively small sample size from a single site, lack of female sports, and non-collision control group limit generalizability of the results. Increasing data consistently suggest sex-related differential response to concussion, with females experiencing greater severity of symptoms and longer recovery time than their male counterparts. Therefore, the data from the current study in male football players unlikely translate into female athletes with subconcussive exposure (e.g., soccer, ice hockey, rugby). Additionally, our findings in tau and DTI/NODDI cannot fully attribute to football-related neural burden, given that we were unable to account for developmental factors and positive effects from exercise due to lack of control group.

We are also aware that the true novelty lies with a longitudinal multimodal relationship, by testing if parameters of neuroimaging and blood biomarkers change over time in relation to head impact exposure. Hirad et al. (70) recently showed longitudinal agreement between DTI and tau, but other biomarkers and NODDI were not included. Therefore, this study is an excellent step to encourage interdisciplinary collaborations between neuroimaging and blood biomarker scientists since these fields rarely intersect to delineate subconcussive brain injury. The potential residual neural burden was accounted for by the number of previous concussions and years of tackle football experience. Although these are commonly used variables, there might be an unquantifiable recall bias in self-reporting. A more rigorous approach would be to use head-impact data from previous seasons and conduct a medical chart review to validate prior concussion history.

## Conclusion

Evidence is beginning to uncover the effects of cumulative concussive and subconcussive head impacts in sports. Neuroimaging and blood biomarkers have been two of the most active areas of research in the neurotrauma community. Our data from DTI/NODDI and blood biomarkers suggest that football players may develop axonal microstructural abnormality particularly in the corpus callosum and surrounding white matter tracts, such as longitudinal fasciculus. Future study is warranted to determine the longitudinal multimodal relationship in response to repetitive exposure to sport-related head impacts.

## Data Availability Statement

The original contributions presented in the study are included in the article/Supplementary Materials, further inquiries can be directed to the corresponding author/s.

## Ethics Statement

The studies involving human participants were reviewed and approved by Indiana University Institutional Review Board. Written informed consent to participate in this study was provided by the participants' legal guardian/next of kin.

## Author Contributions

KKa conceptualized and designed the study, obtained funding, collected data, conducted analysis, drafted the initial manuscript, and reviewed and revised the manuscript. JS and JM designed the study, recruited subjects, collected data, and reviewed and revised the manuscript. MH, MN, and KKe recruited subjects, collected data, reviewed and revised the manuscript. DR conducted follow-up analysis and revised the manuscript. SN and AS contributed to conceptualize the study, obtained funding, reviewed and finalized study protocol, and provided critical review of the manuscript. HC, ZC, and KE designed the study, conducted initial and final analyses, helped draft the manuscript, and reviewed and revised the manuscript. All authors contributed to the article and approved the submitted version.

## Conflict of Interest

The authors declare that the research was conducted in the absence of any commercial or financial relationships that could be construed as a potential conflict of interest.
