# Supplementary material for: Association Between Proteomic Blood Biomarkers and DTI/NODDI Metrics in Adolescent Football Players: A Pilot Study
Source: Front Neurol. 2020 Nov 16;11:581781. doi: 10.3389/fneur.2020.581781 (PMC7701105; doi:10.3389/fneur.2020.581781)
Supplement: Supplementary file 1 [file Table_1.DOCX]

| Supplemental Table 1. Post-hoc correlation results between blood biomarkers and diffusion metrics in the region of interested –highlighted voxels in Fig 1B and the genu of corpus callosum. | | | | | | |
| --- | --- | --- | --- | --- | --- | --- |
|  | MD | FA | AD | RD | ODI | NDI |
| Tau | **0.65 (0.0044)** | -0.22  (0.39) | 0.47  (0.056) | **0.63 (0.0067)** | -0.26  (0.31) | **-0.58 (0.016)** |
| NfL | 0.25  (0.34) | 0.17  (0.51) | 0.29  (0.26) | 0.03  (0.89) | 0  (1) | -0.13  (0.62) |
| GFAP | 0.12  (0.65) | 0.23  (0.28) | 0.19  (0.46) | -0.07  (0.78) | 0.12  (0.64) | 0.18  (0.60) |
| Note. Data shown as Pearson coefficients r (p-values) – **Bold** indicating a significant correlation. The genu of corpus callosum was defined according to the JHU-ICBM white matter labels, which is available in FSL. NfL, neurofilament light. GFAP, glial fibrillary acidic protein. MD, mean diffusivity. FA, Fractional anisotropy. AD, axial diffusivity. RD, radial diffusivity. ODI, orientation dispersion index. NDI, neurite density index. | | | | | | |
